# A cost-effectiveness analysis of a preventive exercise program for patients with advanced head and neck cancer treated with concomitant chemo-radiotherapy

**DOI:** 10.1186/1471-2407-11-475

**Published:** 2011-11-03

**Authors:** Valesca P Retèl, Lisette van der Molen, Frans JM Hilgers, Coen RN Rasch, Annemiek AAMHJ L'Ortye, Lotte MG Steuten, Wim H van Harten

**Affiliations:** 1 Netherlands Cancer Institute-Antoni van Leeuwenhoek Hospital (NKI-AVL), Department of Psychosocial Research and Epidemiology, Plesmanlaan 121, 1066 CX Amsterdam, the Netherlands; 2 Netherlands Cancer Institute-Antoni van Leeuwenhoek Hospital (NKI-AVL), Department of Head and Neck Oncology & Surgery, Plesmanlaan 121, 1066 CX Amsterdam, the Netherlands; 3 Institute of Phonetic Sciences/ACLC, University of Amsterdam, Spuitstraat 210, 1012 VT Amsterdam, The Netherlands; 4 Academic Medical Center, University of Amsterdam, Meibergdreef 9, 1105 AZ Amsterdam, The Netherlands; 5 Netherlands Cancer Institute-Antoni van Leeuwenhoek Hospital (NKI-AVL), Department of Radiation Oncology, Plesmanlaan 121, 1066 CX Amsterdam, the Netherlands; 6 Reade, Centre for Rehabilitation and Rheumatology, location Slotervaart Hospital, Louwesweg 6, 1066 EC Amsterdam, the Netherlands; 7 University of Twente, Department of Health Technology and Services Research, MB-HTSR, PO Box 217, 7500 AE Enschede, the Netherlands

**Keywords:** head and neck cancer, concomitant chemo-radiotherapy, cost-effectiveness, rehabilitation

## Abstract

**Background:**

Concomitant chemo-radiotherapy (CCRT) has become an indispensable organ, but not always function preserving treatment modality for advanced head and neck cancer. To prevent/limit the functional side effects of CCRT, special exercise programs are increasingly explored. This study presents cost-effectiveness analyses of a preventive (swallowing) exercise program (PREP) compared to usual care (UC) from a health care perspective.

**Methods:**

A Markov decision model of PREP versus UC was developed for CCRT in advanced head and neck cancer. Main outcome variables were tube dependency at one-year and number of post-CCRT hospital admission days. Primary outcome was costs per quality adjusted life years (cost/QALY), with an incremental cost-effectiveness ratio (ICER) as outcome parameter. The Expected Value of Perfect Information (EVPI) was calculated to obtain the value of further research.

**Results:**

PREP resulted in less tube dependency (3% and 25%, respectively), and in fewer hospital admission days than UC (3.2 and 4.5 days respectively). Total costs for UC amounted to €41,986 and for PREP to €42,271. Quality adjusted life years for UC amounted to 0.68 and for PREP to 0.77. Based on costs per QALY, PREP has a higher probability of being cost-effective as long as the willingness to pay threshold for 1 additional QALY is at least €3,200/QALY. At the prevailing threshold of €20,000/QALY the probability for PREP being cost-effective compared to UC was 83%. The EVPI demonstrated potential value in undertaking additional research to reduce the existing decision uncertainty.

**Conclusions:**

Based on current evidence, PREP for CCRT in advanced head and neck cancer has the higher probability of being cost-effective when compared to UC. Moreover, the majority of sensitivity analyses produced ICERs that are well below the prevailing willingness to pay threshold for an additional QALY (range from dominance till €45,906/QALY).

## Background

In recent years, concomitant chemo-radiotherapy (CCRT) has become an indispensable organ preserving treatment modality for advanced head and neck cancer, improving local control and overall survival in several anatomical sites [1]. Unfortunately, CCRT can have a detrimental effect on many functions of the upper respiratory and digestive system. Sequellae such as pain, oedema, xerostomia and fibrosis negatively affect mouth opening (trismus), chewing, swallowing and speech [1]. Several studies investigating long-term effects of CCRT have concluded that swallowing and nutritional dysfunction tend to be persistent and can be severe [2-4]. Not surprisingly, therefore, CCRT can have a negative effect on patients' quality of life (QoL) [2]. Moreover, even before onset of treatment patients may already present with pain, impaired swallowing, trismus, aspiration, dietary restrictions and tube dependency, and loss of body weight, because the tumour may disrupt the normal anatomy and thus interfere with normal function [1]. Many studies refer to the importance of rehabilitation after, and even during treatment, in order to support and improve those functions [2]. However, as yet, few studies have investigated the effects of (preventive) rehabilitation exercises on the predictable and inevitable swallowing and mouth opening problems for this patient group. In addition, little is known about the costs and benefits of such exercise programs for head and neck cancer. As the clinical effectiveness is established [4], it is now relevant to embark on cost-effectiveness as a contribution to decision making on coverage.

The aim of this study was to analyze the incremental cost-effectiveness for a preventive exercise program (PREP) versus usual care (UC) for patients with advanced head and neck cancer treated at the Netherlands Cancer Institute - Antoni van Leeuwenhoek Hospital (NKI-AVL).

## Methods

### Case description

To investigate the cost-effectiveness of a preventive (swallowing) exercise program (PREP) compared to usual care (UC) in advanced head and neck cancer, data of two recent clinical trials in the Netherlands Cancer Institute were used [3,4]. In both studies the protocol was approved by the Protocol Review Board of the Netherlands Cancer Institute - Antoni van Leeuwenhoek Hospital (NKI-AVL) and written informed consent was obtained from all patients before entering the study. All patients had advanced (stage III and IV) functional or anatomical inoperable head and neck cancer [5]. All received identical concomitant chemo-radiotherapy (CCRT), which consisted of 100-mg/m^2 ^Cisplatin as a 40 minutes intravenous (IV) infusion on days 1, 22, 43 and combined with radiotherapy, and identical intensive supportive care. Details about patients, methods, and clinical results in both studies have been published previously [3,4,6]. The patient characteristics are summarized in Table 1.

**Table 1:** Patient characteristics of the preceding randomized CCRT trial at in the NKI-AVL of Ackerstaff et al. (usual care) [3] and the randomized CCRT trial at the NKI-AVL of Van der Molen et al. [4] that included a preventive swallowing exercise program (PREP)

	Usual care N = 53	PREP N = 37
**Age in years**		
Median	55	58
Range	24-75	39-77

**Sex**		
Male	36 (68%)	28 (76%)
Female	17 (32%)	9 (24%)

**Stage distribution**		
III	14 (26%)	14 (38%)
IV	39 (74%)	23 (62%)

**Tumour site**		
Oral cavity/oropharynx		16 (43%)
Hypopharynx	42 (79%)	15 (41%)
Nasopharynx	11 (21%)	6 (16%)

**Follow up**		
	Pre	Pre
	7 wks	10 wks
	1-year	1-year

**Tube dependency**		
before CCRT	8 (15%)	0 (0%)
1-year after CCRT	13/53 (25%)	1/37 (3%)

**Aspiration at 1-year**	Unknown	1/37 (3%)

Hospital admission days after completion of CCRT (mean per patient/year)	4.49	3.19

Single day admissions after completion of CCRT (mean per patient/year)	0.70	0.16

The UC data are derived from a multi-center randomized controlled trial (RCT), comparing intra-arterial (IA) and intravenous (IV) chemo radiation in advanced head and neck cancer [3]. Only the data of the 53 patients treated at the NKI-AVL, randomized in the IV arm, and still alive and disease free at 12 months were analysed for this cost-effectiveness study.

The PREP data are derived from a clinical trial conducted immediately following the former RCT. In this second RCT the effects of preventive strength and stretch exercises on (long-term) swallowing and/or mouth opening problems caused by CCRT, as an adjunct to UC, were assessed in 55 advanced head and neck cancer patients [4]. Before treatment all patients were randomized into two groups: an experimental group that was provided with the TheraBite^® ^Jaw Motion Rehabilitation System™ and a group receiving standard intervention (Standard group). The rationale and a detailed description of the exercises have been published previously [4]. In short, both regimes consist of comparable stretch and strength exercises to keep the swallowing musculature active before, during, and after CCRT, even when patients are not swallowing because of (naso) gastric tube feeding. Patients were encouraged to practice 3 times a day and to integrate the exercises into other daily activities at home. Participants were provided with verbal and written instructions prior to treatment, at which time they also started practicing, thus, when oral intake was not yet influenced by the treatment. Thirty-seven of the 55 included patients were still alive and disease free at 12-months. Since no significant differences in QoL, costs and functional outcomes were found between the two arms, for the present cost-effectiveness study both PREP arms were taken together [4].

The main outcome variables of interest for this cost effectiveness assessment were tube dependency at 12 months, and number of days patients were admitted to the hospital after completion of the CCRT in the first year. In the UC cohort, tube dependency was 13/53 (25%), and in the PREP cohort 1/37 (3%). The mean number of extra admission days in the hospital post-CCRT was 4.5 (SE 2.8) in the UC, and 3.2 (SE 1.2) in the PREP cohort.

### Model description

A Markov decision model was developed to compare the PREP versus UC for advanced head and neck cancer. The model was constructed with three mutually exclusive health states: "complete remission", "recurrent disease" and "death" (death of cancer or other causes). The input regarding treatment success rates, and probability of recurrence were based on the published outcome data from our institute [6]. We assumed that the PREP has no direct influence on survival [7-9]. Input on aspiration for UC was based on the empirical data, for PREP the value was assumed, based on the literature. The input of feeding substitutes and hospitalization were based on the above-described databases: the series of Ackerstaff et al. [3], as UC and that of Van der Molen et al. [4], as the PREP strategy. The model simulated the course of events in a hypothetical cohort of 1000 patients aged 55 years with a stage III or IV squamous cell carcinoma of the head and neck treated at the NKI-AVL. Possible complications from the treatment were modelled up to 1 year from the start of treatment. The cycle length of the model was one month, with a total simulated time horizon of 1 year. The analysis was performed from the health care perspective of the NKI-AVL. All costs were reported in year 2008 Euros (Table 2).

**Table 2:** Input Parameters of base case and sensitivity analysis, including days of feeding substitutes, treatment success rates, aspiration probabilities, utilities and costs

Parameter	Mean	SE	Distribution	Source
**Care**				
Days FS RB 2 months	0.760^a^			4
Days FS UC 2 months	0.820			3
Days FS RB 3 months	0.370			4
Days FS UC 3 months	0.700			3
Days FS RB 12 months	0.030			4
Days FS UC 12 months	0.240			3

**Success rates**				
CCRT	0.940^b^	0.030	Beta	5
Recurrence rates	0.012^c^	0.010	Beta	5
Aspiration PREP	0,027	0.015	Beta	4
Aspiration UC	0,054	0.015	Beta	Assumption

**Utilities**				
During CCRT PREP	0.617	0.015	Beta	Assumption
During CCRT UC	0.517	0.015	Beta	3
Cured PREP	0.854	0.015	Beta	Assumption
Cured UC	0.754	0.015	Beta	3
Recurrent disease	0.517	0.015	Beta	Assumption

**Costs**				
Hospital days NKI	€ 476	Fixed		8
Day care NKI	€ 229	Fixed		8
Feeding substitutes	€ 845	Fixed		NKI-AVL
Professional Tariff	€ 3,252	Fixed		DBC-system
CCRT	€ 31,000	Fixed		NKI-AVL
Palliative care	€ 30,000	Fixed		Assumption
Pneumonia	€ 1,904	Fixed		3, 4, 7

**Sensitivity analysis**				
*Utilities*				
During CCRT PREP low	0.567	0.015	Beta	Assumption
During CCRT PREP high	0.667	0.015	Beta	Assumption
Cured PREP low	0.804	0.015	Beta	Assumption
Cured PREP high	0.904	0.015	Beta	Assumption
*Costs*				
Professional Tariff low	€ 1,214	Fixed		DBC-system
Professional Tariff high	€ 7,058	Fixed		DBC-system

PREP = preventive exercise program

UC = usual care

FS = feeding substitutes

NKI = Netherlands Cancer Institute

CCRT = concomitant chemo-radiotherapy

FS = Feeding substitutes

SE = Standard deviation

^a ^calculated to monthly rate

^b ^progression free survival probability of 50% over 5 years calculated to monthly survival rate

^c ^recurrence rate from 'complete remission' to recurrent disease

### Costs

In the NKI-AVL the costs for treatment where measured by means of clinical pathways that patients followed when receiving CCRT. Besides treatment costs, feeding substitutes, pneumonia as adverse event and hospital days were derived from the NKI-AVL hospital charts and administration. The professional costs of PREP were derived from the Dutch Diagnosis Treatment Combination (DBC) "DBC-system" list, this tariff includes all possible involved disciplines in the PREP (19-49 hours for €3,252). Use of feeding substitutes (tube feeding) was calculated per disease severity stage from the two databases. It was assumed that from the patients needing tube feeding, 50% received a nasal tube and 50% received a gastronomy-tube (Table 2).

### Health effects

The quality of life of patients treated with CCRT was examined by Ackerstaff et al. [3]. For UC during treatment the QoL result of 7 weeks was incorporated (0.517), for UC after treatment, the QoL result of 12 months was taken (0.754) [3]. Assumptions as to how these results would be influenced by the PREP were based on published literature and informal expert elicitation (Table 2).

### Analysis

We programmed the model in Microsoft Excel (Microsoft, Redmond, WA) and validated it using various sensitivity analyses. Future costs and effects were discounted to their present value by a rate of 4% and 1.5% per year respectively, according to Dutch guidelines [10]. Incremental cost-effectiveness ratios (ICERS) were calculated by dividing the incremental costs by incremental quality adjusted life years (QALYs). Stochastic uncertainty in the input parameters was handled probabilistically, by assigning distributions to parameters (Table 2) [11]. Parameter values were drawn randomly from the assigned distributions, using Monte Carlo simulation with 1000 iterations. The results of the simulation of the hypothetical cohort of 1000 patients are illustrated in a Cost-Effectiveness (CE) plane, each quadrant indicates whether a strategy is more or less expensive and more or less effective [12]. Cost-effectiveness acceptability curves (CEACs) to show decision uncertainty are presented. CEACs show the probability that a pathway has the highest net monetary benefit, and thus is deemed cost-effective, for a range of Willingness to Pay (λ) values for one additional QALY, also referred as the ceiling ratio. This definition involves a Bayesian definition of probability i.e. the probability that the hypothesis ('PREP is cost-effective compared to UC') is true given the data. The two curves therefore always sum to 100% for one given value of λ [13]. In the Netherlands an informal ceiling ratio of €80,000 per QALY exists (Dutch Council for Public Health and Health Care 2006), and for preventive care programs of €20,000 per QALY. The National Institute for Health and Clinical Excellence in the United Kingdom uses a general ceiling ratio between £20,000-£30,000 per QALY. In this analysis, we use the Dutch threshold for preventive care programs, €20,000 per QALY.

### Sensitivity Analyses

We performed four one-way sensitivity analyses. The first two sensitivity analyses tested the robustness of the model outcomes against changes in the utility estimates (i.e. higher and lower estimates), as the current estimates are preliminary. For the two other sensitivity analyses, lower and higher cost estimates (€1,213 and €7,058) were imported for the resource use associated with paramedical care delivery in the rehabilitation program (for respectively 7-18 hours or 50-129 hours). In addition, we performed two two-way sensitivity analyses to test the most uncertain parameters, such as a variation in utilities in combination with the various DBC tariffs, and the variation of utilities in combination with the probability of aspiration.

For various scenarios regarding costs and QALYs, we also present the findings as cost-effectiveness acceptability frontiers that illustrate the probability of any intervention being optimal compared to its alternative. The optimal intervention is defined as the one with the highest expected net health benefit. Each cost-effectiveness frontier also illustrates the potential crossover when one intervention is substituted by another as the one with the highest probability of being optimal, and therefore provides useful information for policy makers.

### Expected value of perfect information (EVPI)

Uncertainty in the cost-effectiveness results was also presented and used to inform future research priorities using Value of information analysis (VOI) analyses based on the expected value of perfect information (EVPI). VOI can be used to support decisions on focus and design of further research, assuming that additional evidence on the relevant aspects can be desired, but that the amount and specific requirements for further research will depend on the parameters which are causing the most uncertainty [14]. Generally, information is valuable when there is great uncertainty surrounding a decision and when that decision likely affects a large number of people in a meaningful way. If one had perfect information about the risks and benefits of a particular technology, decision makers in theory would always be able to make correct choices regarding the use of the technology. The difference between the expected net benefit obtained using perfect information and the expected net benefit obtained in the presence of uncertainty (that is, the maximum expected net benefit obtained with less than perfect information) is known as the expected value of perfect information (EVPI). It can be interpreted as the maximum amount the decision maker would be willing to spend to obtain perfect information [15].

## Results

### Mean results

The total health care costs (treatment costs + preventive exercises) per patient were: €42,271 for the preventive exercise program (PREP), and €41,986 for usual care (UC). The quality adjusted life years amounted to: 0.77 (PREP), and 0.68 (UC). The difference in costs per QALY of the PREP strategy compared to the UC strategy amounted to €285 (= 0.7% of the total treatment costs). In comparison to UC, the PREP for advanced head and neck tumours costs €3,197 per QALY gained and was found to be more effective and slightly more costly (Table 3).

**Table 3:** Results of the base case analysis; incremental (difference) in QALYs, incremental costs and the incremental cost-effectiveness ratio (ICER) of the comparison between Usual Care and PREP

	Costs	QALYs	Incremental COSTS	Incremental QALYS	ICER Costs/QALY
**PREP**	€42,271	0.77	€285	0.09	€3,197*
**Usual Care**	€41,986	0.68			

*The numbers might not add up to 100% because of rounding; 284.8849/0.0891 = 3197.3614

### Uncertainty Analyses

**Figure 1:**
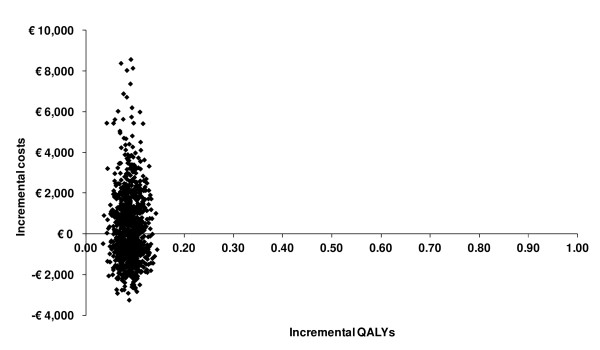
**Cost-Effectiveness plane; scatter plot showing the mean differences in costs and outcomes from the data using 1000 bootstrap replicates**.

**Figure 2:**
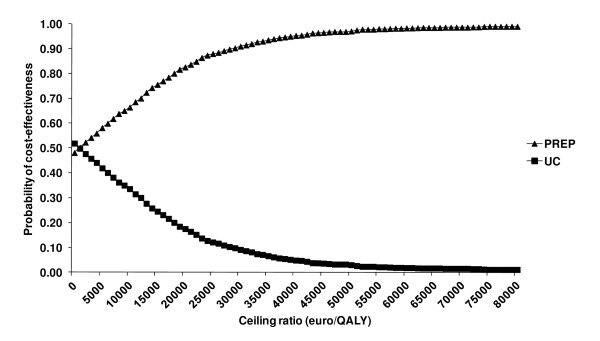
**Cost-effectiveness Acceptability Curves (CEAC); presenting the probability that the PREP is cost-effective compared with the Usual Care for a range of values of thresholds (ceiling ratios, willingness to pay for one QALY)**.

When focusing on quality adjusted survival, the PREP has a higher probability of being cost-effective compared to UC, as long as the willingness to pay threshold for 1 additional QALY is at least €3,200/QALY (Figure 1 and 2). At the prevailing threshold of €20,000/QALY the probability for PREP being cost-effective compared to UC was 83%.

### Sensitivity analyses

**Figure 3:**
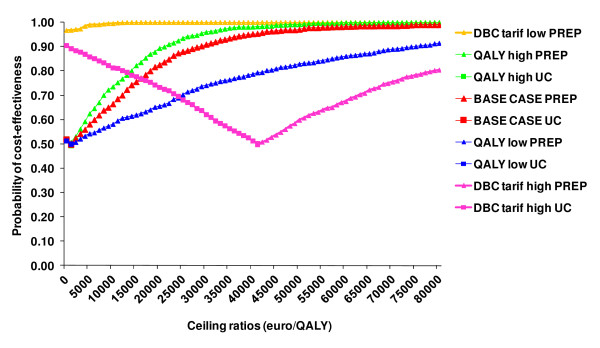
**CEAC-frontiers; plotting the extent of uncertainty associated with the optimal strategy**.

The sensitivity analysis using lower utility estimates resulted in an incremental cost-effectiveness ratio (ICER) of €6,393/QALY; higher utilities resulted in an ICER of €2,131/QALY. The sensitivity analysis considering a lower resource use (fewer hours of professionals) resulted in UC being dominated by PREP, i.e. PREP is more effective and less costly than UC. Modelling a higher resource use (more hours) for the PREP resulted in a higher ICER of €45,906/QALY (Figure 3). The results of the two-way sensitivity analyses are listed in Table 4, the majority of the analyses resulted with an ICER below the ceiling ratio of €20,000/QALY.

**Table 4:** Results of the two-way sensitivity analysis; range of variables of utilities versus different DBC tariffs and range of variables of utilities versus different aspiration probabilities

Utilities	0.80	0.85	0.90
DBC tariffs			
**€1,214**	-€ 39,349	-€ 19,674	-€ 13,116
**€3,252**	€ 6,394	**€3,197**	€ 2,131
**€7,058**	€ 91,814	€ 45,907	€ 30,605

**Utilities**	**0.80**	**0.85**	**0.90**
**aspiration**			

**0.02**	€ 23,442	€ 11,721	€ 7,814
**0.04**	€ 13,430	€ 6,715	€ 4,477
**0.06**	€ 3,417	€ 1,709	€ 1,139

### Expected value of information (EVPI)

**Figure 4:**
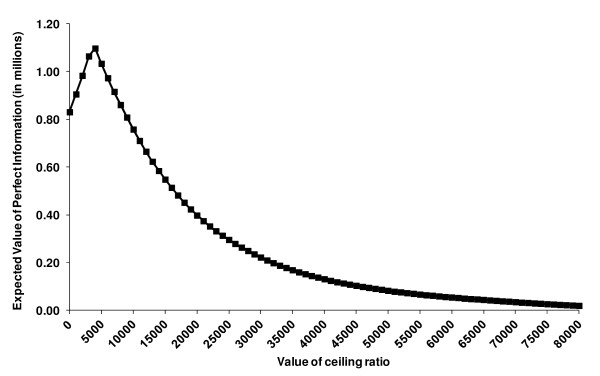
**Expected Value of Perfect Information (EVPI) for the population, base case analysis**.

At a ceiling ratio of €20,000/QALY the probability for PREP being cost-effective compared to UC was 83%, which shows a considerable decision uncertainty using the current available data. The EVPI for the base case resulted in €398,063, providing the upper boundary for investing research funds in further clinical trials to obtain perfect information on the cost-effectiveness of PREP versus UC. The EVPI demonstrated potential value in undertaking additional research to reduce the existing decision uncertainty (Figure 4).

## Discussion

This cost-effectives analysis, as based on two patient populations with advanced head and neck cancer treated with CCRT of which one was additionally treated with a preventive exercise program (PREP) to prevent or limit the functional side effects of the treatment, and one receiving no additional care, showed that quality-adjusted survival was higher for the PREP. When focusing on quality adjusted survival, PREP has an 83% probability of being cost-effective at the prevailing threshold of €20,000/QALY.

Because the presented results are based on preliminary data, assumptions had to be made regarding the utilities and no empirical data was yet available regarding decrease of e.g. aspiration, which can be a life threatening problem in case of severe swallowing problems resulting from CCRT. However, a quarter of the patients in the usual care cohort needed a feeding tube at 12 months, in contrast to only 3% in the PREP cohort. This suggests that patients in the UC group are more likely to suffer from aspiration than those in the PREP, something worthwhile to look into in more detail in future studies. Furthermore, not all relevant improvements can easily be expressed in costs or utilities and this may be even more difficult because of the various different effects that rehabilitation may have. E.g., it is likely that the assumptions regarding the incremental utility of the PREP and the probability of aspiration are an underestimation, because patients' functional improvement is likely to become even better when patients are receiving the full rehabilitation program. This is partly covered by the sensitivity analyses but may also be investigated in more detail with a Contingent Valuation study, where someone's willingness to pay for improvements in specific aspects of quality of life can be assessed.

Another limitation of this cost-effective analysis is that the patient distribution in the two cohorts is not completely comparable. Although all patients had stage III or IV disease, the distribution according to stage (more stage IV in the UC group) and anatomical site (no oral cavity/oropharynx in the UC group) was somewhat different. Next to this, the chemotherapy type and scheme in both cohorts was identical except with respect to the application of radiotherapy. In the IV arm of the study of Ackerstaff et al. [3], roughly one fourth of the patient population was treated with intensity-modulated radiotherapy (IMRT), whereas in all patients in the study of Van der Molen et al. [4] IMRT was applied. These differences could have influenced the functional outcomes in the two cohorts. Exact data about these aspects unfortunately are not retrievable. But we are sufficiently confident, that in the study of Ackerstaff et al. [3] most of the patients that were disease free at 1-year, indeed received IMRT.

Literature suggests that other functions will also improve as a result of a PREP, and thus the cost-effectiveness would most likely improve even more when these other functions are taken into account [16-22]. Model inputs for UC were based on a former study performed in the Netherlands Cancer Institute (NKI-AVL) [3], to be able to make a clear comparison. However, the NKI-AVL, as a specialized tertiary care hospital, is a pioneer in this field and thereby already had implemented some rehabilitation components in the UC-series of Ackerstaff et al. [3], e.g. not 'automatically' providing a feeding tube at the onset of treatment, but trying with intensified support to maintain oral feeding for as long as possible. If the comparison was made with the care as provided by the national guidelines at that time, the analysis would probably result in an even more favourable ICER.

Because of the promising results of this PREP, a more comprehensive head and neck rehabilitation program has been developed to stimulate participation in everyday life activities with all the pathophysiological or anatomical changes and restrictions accompanying head and neck cancer taken into account. To achieve this, the NKI-AVL is cooperating with the rehabilitation centre Amsterdam (READE) to accomplish this comprehensive rehabilitation program based on the 'International Classification of Functioning, Disability and Health' (ICF) core sets for head and neck cancer [23]. It is conceivable that such a more intensified program can boost rehabilitation results even further and research as to that is planned.

This cost-effectiveness analysis is based on a Markov decision model because this allows synthesizing data from various sources, when an empirical, longer term, head to head trial of PREP versus UC has not yet been performed. As with all modelling studies that extrapolate data beyond the time horizon of a clinical trial, the outcomes have to be interpreted as expected costs and outcomes, based on the best available current evidence.

## Conclusion

This study shows that, based on current available evidence, the addition of a preventive (swallowing) exercise program to concomitant chemo-radiotherapy in advanced head and neck cancer improves quality-adjusted survival and has a higher probability of being cost-effective compared to usual care. The calculated additional costs of €3,200/QALY is well below the threshold of €20,000/QALY, which currently is handled for preventive care programs e.g. by the Dutch Council for Public Health and Health Care and the National Institute for Health and Clinical Excellence in the United Kingdom. With a relatively low additional investment in research the uncertainty in the calculation of the cost effectiveness can be considerably improved, which is currently ongoing.

## Competing interests

The authors declare that they have no competing interests.

## Authors' contributions

VR performed the cost-effectiveness analysis, carried out the acquisition of the data, and drafted the manuscript. LvdM carried out the acquisition of the data, made substantial contribution to the analysis and co-drafted the manuscript. FH has made substantial contributions to the conception and design to the study and revised the manuscript critically. CR and AO have made substantial contributions to the critical revision of the manuscript. LS participated in the design of the study and made substantial contribution to the cost-effectiveness analysis. WvH participated in its conception and design and coordination and helped to draft the manuscript. All authors read and approved the final manuscript.

## Pre-publication history

The pre-publication history for this paper can be accessed here:

http://www.biomedcentral.com/1471-2407/11/475/prepub

## References

[B1] Van der Molen L van Rossum MA Ackerstaff AH Smeele LE Rasch CR Hilgers FJ Pretreatment organ function in patients with advanced head and neck cancer: clinical outcomes measures and patient' views BMC Ear Nose Throat Disord 2009 9 10 10.1186/1472-6815-9-10 19912667 PMC2779790

[B2] Van der Molen L van Rossum MA Burkhead LM Smeele LE Hilgers FJ Functional outcomes and rehabilitation strategies in patients treated with chemoradiotherapy for advanced head and neck cancer: a systematic review Eur Arch Otorhinolaryngol 2009 266 6 901 2 10.1007/s00405-008-0845-z 18825400

[B3] Ackerstaff AH Balm AJ Rasch CR First year quality-of-life assessment of an intra-arterial (RADPLAT) versus intravenous chemoradiation phase III trial Head Neck 2009 31 1 77 84 10.1002/hed.20937 18972429

[B4] Van der Molen L van Rossum MA Burkhead LM Smeele LE Rasch CR Hilgers FJ A Randomized Preventive Rehabilitation Trial in Advanced Head and Neck Cancer Patients Treated with Chemoradiotherapy: Feasibility, Compliance and short-term effects Dysphagia 2010 10.1007/s00455-010-9288-y PMC3098976 20623305

[B5] Kreeft A Tan IB van den Brekel MW Hilgers FJ Balm AJ The surgical dilemma of 'functional inoperability' in oral and oropharyngeal cancer: current consensus on operability with regard to functional results Clin Otolaryngol 2009 34 2 140 6 10.1111/j.1749-4486.2009.01884.x 19413612

[B6] Rasch CR Hauptmann M Schornagel J Intra-arterial versus intravenous chemoradiation for advanced head and neck cancer- Results of a randomized phase 3 trial Cancer 2010 116 9 2159 65 20187094 10.1002/cncr.24916

[B7] Bernier J Domenge C Ozsahin M Postoperative irradiation with or without concomitant chemotherapy for locally advanced head and neck cancer N Engl J Med 2004 350 19 1945 52 10.1056/NEJMoa032641 15128894

[B8] Nguyen NP Frank C Moltz CC Analysis of factors influencing aspiration risk following chemoradiation for oropharyngeal cancer Br J Radiol 2009 82 980 675 80 10.1259/bjr/72852974 19332514

[B9] Langerman A MacCracken E Kasza K Aspiration in Chemoradiated patients with head and neck cancer Arch Otolaryngol head and neck surg 2007 133 12 1289 1295 10.1001/archotol.133.12.1289 18086974

[B10] Oostenbrink JB Koopmanschap MA Rutten FFH Handleiding voor kostenonderzoek, methoden en richtlijnprijzen voor economische evaluaties in de gezondheidszorg 2000 Amstelveen: www.cvz.nl Health Care Insurance Board

[B11] Weinstein MC Recent Developments in Decision-Analytic Modelling for Economic Evaluation Pharmacoeconomics 2006 24 1043 1053 10.2165/00019053-200624110-00002 17067190

[B12] Fenwick E Claxton K Sculpher M Representing uncertainty: the role of cost-effectiveness acceptability curves Health Econ 2001 10 779 787 10.1002/hec.635 11747057

[B13] Briggs A Claxton K Sculpher M Decicion Modelling for Health Economic Evaluation 2006 Oxford University Press

[B14] Vallejo-Torres L Steuten LM Buxton MJ Integrating health economics modeling in the product development cycle of medical deviced: a Bayesian approach Int J Technol Assess Health Care 2008 24 4 459 64 18828941 10.1017/S0266462308080604

[B15] Willan AR Pinto EM The value of information and optimal clinical trial design Stat Med 2005 24 1791 1806 10.1002/sim.2069 15806619

[B16] Burkhead LM Sapienza CM Rosenbek JC Strength-training exercise in dysphagia rehabilitation: principles, procedures, and directions for future research Dysphagia 2007 22 3 251 65 10.1007/s00455-006-9074-z 17457549

[B17] Kubrak C Olson K Jha N Jensen L McCargar L Seikaly H Nutrition impact symptoms: Key determinants of reduced dietary intake, weight loss, and reduced functional capacity of patients with head and neck cancer before treatment Head Neck 2009 10.1002/hed.21174 19626639

[B18] Lazarus CL Logemann JA Pauloski BR Rademaker AW Larson CR Mittal BB Swallowing and tongue function following treatment for oral and oropharyngeal cancer J Speech Lang Hear Res 2000 43 4 1011 23 11386468 10.1044/jslhr.4304.1011

[B19] List MA Bilir SP Functional outcomes in head and neck cancer Semin Radiat Oncol 2004 14 2 178 89 10.1053/j.semradonc.2003.12.008 15095263

[B20] Reilly JJ Does nutrition management benefit the head and neck cancer patient? Oncology (Williston Park) 1990 4 6 105 15 2144997

[B21] Rosenthal EL Lindeboom JA Eisbruch A Prevention and treatment of dysphagia and aspiration after chemoradiation for head and neck cancer Journal of clinical oncology 2006 24 17 2636 43 10.1200/JCO.2006.06.0079 16763277

[B22] Salerno G Cavaliere M Foglia A Pellicoro DP Mottola G Nardone M The 11th nerve syndrome in functional neck dissection Laryngoscope 2002 112 7 Pt 1 1299 307 12169917 10.1097/00005537-200207000-00029

[B23] World Health Organization Dutch translation of 'International Classification of Functioning, Disability and Health: ICF' 2001 Geneva: WHO

